# The role of circadian rhythm-related genes in type 2 diabetes from a multi-omics perspective

**DOI:** 10.7189/jogh.15.04227

**Published:** 2025-08-29

**Authors:** Liangxiao Xie, Duobin Huang, Xiaoyun Zha, Changshun Wei, Jiajia Dong, Huaqiang Zheng, Zehong Xu, Jinzhi Wu, Pengbin Lai

**Affiliations:** Zhangzhou Affiliated Hospital of Fujian Medical University, Department of Endocrinology and Metabolism, Zhangzhou, China

## Abstract

**Background:**

Recent studies have established a connection between circadian rhythm disruption and the development of type 2 diabetes mellitus (T2DM), yet the underlying genetic mechanisms remain inadequately understood. This research aims to elucidate the causal relationships between circadian rhythm-related genes and T2DM by utilising a multi-omics approach.

**Methods:**

The study employed the GeneCards database to identify genes associated with circadian rhythms. Integration of quantitative trait loci (QTL) data for gene expression (eQTLs), DNA methylation (mQTLs), and protein expression (pQTLs) was conducted alongside genome-wide association study summary data for T2DM sourced from the Integrative Epidemiology Unit (IEU) and FinnGen databases.

**Results:**

The summary-databased Mendelian randomization (SMR) methodology was utilised to analyse potential causal relationships, supported by colocalisation analyses to confirm the origin of genetic signals. The analysis yielded 47 mQTLs, five eQTLs, and three pQTLs significantly correlated with T2DM outcomes, maintaining a significance threshold of *P*_SMR_multi <0.05, *P*_SMR<0.05, and *P*-HEIDI>0.01.

**Conclusions:**

The integration of mQTL and eQTL data identified 20 critical methylation sites and 13 associated genes relevant to T2DM, with Heat Shock Factor 1(HSF1) emerging as a pivotal gene. Notably, high methylation at the site cg18814314 was inversely correlated with T2DM risk, while increased expression of HSF1 showed a positive correlation. These findings suggest that HSF1 plays a vital role in the pathogenesis of T2DM through circadian rhythm regulation, highlighting its potential as a target for early intervention strategies. Further research is warranted to investigate the timing of circadian gene expression and methylation in T2DM development.

Type 2 Diabetes Mellitus (T2DM) is a chronic metabolic disorder characterised by high blood glucose levels, and its prevalence has been steadily increasing globally in recent years, posing a significant threat to public health [1,2]. According to the International Diabetes Federation, approximately 463 million adults worldwide are affected by diabetes, with over 90% of them having T2DM [3,4]. The clinical features of T2DM primarily include insulin resistance and dysfunction of pancreatic β-cells, leading to abnormal blood glucose regulation [5]. Long-term hyperglycaemia can lead to various complications such as cardiovascular diseases, kidney disease, retinopathy, and neuropathy [6]. Understanding the pathogenesis of T2DM is crucial for developing effective prevention and treatment strategies.

Circadian rhythm, as an intrinsic biological clock system, is jointly regulated by endogenous rhythms and external environmental factors [7]. In mammals, the circadian rhythm is controlled by the Suprachiasmatic Nucleus in the hypothalamus, which maintains rhythmic stability through the Transcription-Translation Feedback Loop [8]. Studies have shown that circadian rhythm has a significant impact on metabolic functions, and its disruption is closely associated with obesity, metabolic syndrome, and the development of T2DM [9,10]. This effect may be mediated through the rhythmic regulation of metabolic genes and the oscillations in insulin sensitivity and secretion [11]. Furthermore, disturbances in circadian rhythm can impair blood glucose regulation, leading to insulin resistance and dysfunction of pancreatic β-cells, ultimately affecting glucose levels [12,13]. Research also indicates that circadian rhythm disruption is closely linked to inflammatory responses, lipid metabolism, and energy balance regulation, further contributing to the development and progression of T2DM [14,15]. Although these studies have revealed potential associations between circadian rhythm and T2DM, the specific mechanisms and pathogenic pathways still require further investigation.

Summary-databased Mendelian randomization (SMR), which combines genome-wide association study (GWAS) data and molecular quantitative trait loci data – such as gene methylation quantitative trait loci (mQTL), gene expression quantitative trait loci (eQTL), and protein quantitative trait loci (pQTL) – enables the identification of target genes linked to T2DM risk variants through causal inference [16]. By applying heterogeneity-independent instruments (HEIDI) test, researchers can identify those multiple association signals that may be caused by the same functional variant and distinguish them from chance-associated variants [17]. This helps to more accurately localise true functional variants and provide insight into their influences on T2DM. For example, one study identified several potential genes associated with atrial fibrillation through a combination of GWAS, eQTL studies and mQTL studies [18]. Furthermore, SMR analysis has demonstrated its robust analytical capabilities in identifying disease biomarkers[19]. This innovative approach not only enhances the statistical strength of causal inference but also offers a comprehensive perspective by exploring the interactions between QTLs and T2DM across various molecular levels.

In this study, we utilised SMR to identify potential causal genes associated with T2DM risk and further understand the underlying genetic mechanisms.

Although the association between circadian rhythm disorders and T2DM has been widely reported, the specific causative circadian rhythm-related genes and their potential regulatory mechanisms at the epigenetic, transcriptomic, and proteomic levels remain unclear. Therefore, this study aims to utilise SMR methods, integrating QTL data from multiple omics (including methylation, expression, and protein quantification) with large-scale T2DM GWAS summary statistics, to systematically identify circadian rhythm genes with potential causal relationships to T2DM risk and explore the biological pathways they may be involved in. Through this approach, we hope to reveal the roles of new candidate genes in the cross-network of circadian rhythm and metabolic regulation, thereby providing multi-dimensional genetic evidence and functional clues for understanding the pathogenesis of T2DM.

## METHODS

### Study design

**Figure 1:**
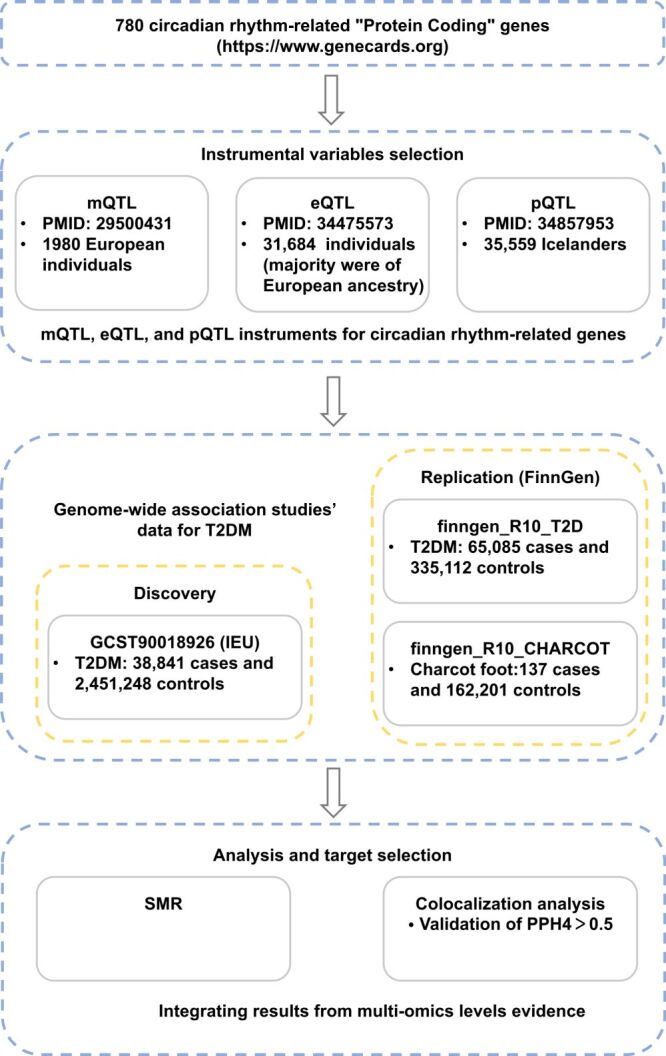
Flowchart of the analyses performed.

In this study, we extracted instrumental variables for circadian rhythm-related genes at the levels of methylation, gene expression, and protein abundance. Subsequent independent Mendelian randomization (MR) analyses were conducted for T2DM at each biological level. The Integrative Epidemiology Unit (IEU) data set was used as the primary discovery data set [20]. To validate our findings, we utilised the FinnGen study data set for replication analysis [21]. To strengthen causal inference, colocalisation analyses were further applied. By integrating results from these three distinct levels of MR analyses, we identified causal candidate genes. There is no overlap in samples between the exposure and outcome populations. The design of the present study and the workflow of the selection of genetic variants and analytical methods are shown in **Figure 1**.

### Data resources

In this study, we downloaded circadian rhythm-related genes from the GeneCards database[22] using the keyword ‘circadian clock’ and identified a total of 780 circadian rhythm-related protein-coding genes. Meanwhile, from the initially identified 780 protein-coding genes, we selected those that had corresponding QTL data in the selected blood-based eQTL, mQTL and pQTL data sets for subsequent analysis.

Genome-wide association study (GWAS) summary statistics for T2DM were obtained from the IEU data set, which included 38 841 cases and 2 451 248 controls. For the replication stage, we used the FinnGen study data set, which included 65 085 cases and 335 112 controls for T2DM, and 137 cases and 162 201 controls for charcot foot (Table S1 in the **Online Supplementary Document**). The IEU data set integrates multi-source cohort study data, including a wide geographical distribution and diverse ethnic groups of the included population, covering people from Europe, Asia, America and other regions. The European population accounts for a large proportion, and there is also a certain proportion of Asian, Native American and other ethnic groups. The sample age range is wide, covering all age groups. The FinnGen study is based on the Finnish population, with samples drawn from multiple cohorts across Finland, including both healthy individuals and patients. The Finnish population has a unique genetic background, and the study has recorded detailed information about the participants. The sample sources of the two projects are independent of each other, and the currently available GWAS summary statistics do not show a significant overlap ratio of samples. Blood eQTLs data for circadian clock genes were obtained from eQTLGen, which includes genetic data on blood gene expression from 31 684 individuals [23]. Blood mQTLs summary data were derived from a meta-analysis of two European cohorts: the Brisbane Systems Genetics Study (n = 614) and the Lothian Birth Cohorts (n = 1366) [24]. Summary statistics for genetic associations with circulating protein levels were extracted from a pQTLs study by Ferkingstad et al., which included 35 559 Icelanders [25]. All summary statistics used in the MR analyses were generated by previous publicly available studies, all of which had obtained ethical approval.

### Summary-databased MR analysis

We used SMR to estimate the association of circadian rhythm-related gene methylation, expression, and protein abundance with T2DM. When exposure and outcome are derived from two independent cohorts with large sample sizes, SMR can achieve higher statistical power than traditional MR analysis based on the top associated *cis*-quantitative trait loci (*cis*-QTLs) [26]. In this study, the most significant cis-QTLs were selected using a window centred around the corresponding gene (±1000 kb) and a *P*-value threshold of 5.0 × 10 − 8 [27]. Single nucleotide polymorphism (SNP) with allele frequency differences exceeding a specified threshold (set at 0.2 in this study) in any pairwise data sets (including the linkage disequilibrium reference sample, QTL summary data, and outcome summary data) were excluded. For eQTL, mQTL, and pQTL, the default proportion of SNPs with allowable allele frequency differences was set at 0.05 [28]. This restriction aims to further control the potential impact that may arise from the mismatch of allele frequencies, ensuring the robustness of the research results. Through the above strict allele frequency matching checks and restriction measures, we have ensured that the genetic variations used in the study have a good consistency in allele frequencies, thereby enhancing the reliability of the MR analysis results.

We explored the causal relationship between mQTLs and eQTLs by treating mQTLs as the exposure and eQTLs as the outcome. Building on SMR analysis, we employed a multi-SNP SMR analysis method (smr-multi) to consider multiple SNPs at QTLs loci in SMR analysis [26]. This study focuses on the results obtained from this method. Subsequently, the HEIDI test was used to distinguish pleiotropy from linkage, with *P*-HEIDI>0.01 indicating the absence of pleiotropy [29]. Additionally, we adjusted the *P*-values from SMR analysis using the Benjamini-Hochberg method and considered associations with false discovery rate (FDR) corrected *P*-values <0.05 and *P*-HEIDI>0.01 for eQTLs and mQTLs colocalisation analysis. To control the risk of false positives brought about by multiple testing, we adopted the Benjamini-Hochberg method to adjust the *P*-values in the SMR analysis. This method, by controlling the FDR, can to a certain extent reduce the false positive rate and enhance the credibility of the results. The SMR and HEIDI tests were implemented using the SMR software tool, SMR version 1.3.1 (University of Queensland, Brisbane, Australia) [24].

### Colocalisation analysis

We conducted colocalisation analysis using the coloc *R* package to detect shared causal variants between T2DM and the identified circadian rhythm-related mQTLs, eQTLs, or pQTLs. In colocalisation analysis, five different posterior probabilities are reported, corresponding to five exclusive hypotheses:

1. no genetic association with SNPs for either trait (H0)

2. genetic association with SNPs exists only for gene expression (H1)

3. genetic association with SNPs exists only for disease risk (H2)

4. both traits are associated with SNPs, but with distinct causal variants (H3)

5. both traits share the same causal variants (H4).

According to published literature, we performed colocalisation analysis on SNPs in the top QTLs colocalisation region windows (±1000 kb upstream and downstream) [30,31]. The default prior probability for SNPs being associated with both the exposure and the outcome is *P*12 = 1 × 10 − 5 [32]. Although a posterior probability of colocalisation (PPH4)≥0.8 has been shown to indicate strong Bayesian evidence supporting colocalisation, Michael S. Breen et al. observed that many loci with PPH4 ≥ 0.5 also appear qualitatively consistent with colocalisation phenomena [33,34]. Therefore, to enhance the sensitivity of our colocalisation analysis and ensure the capture of more potential colocalisation signals, especially since we are in an exploratory phase of the study, we chose PPH4 > 0.5 as the posterior probability threshold for colocalisation. This can provide more comprehensive preliminary clues.

All statistical analyses were performed using *R*, version 4.3.0 (R Foundation for Statistical Computing, Vienna, Austria). The *R* package ‘CMplot’ was used for Manhattan plot generation, and ‘forestplot’ was used for forest plot generation. Code for SMRLocusPlot and SMREffectPlot was sourced from Zhu et al. [26].

## RESULTS

### Circadian rhythm-related gene methylation and T2DM

**Figure 2:**
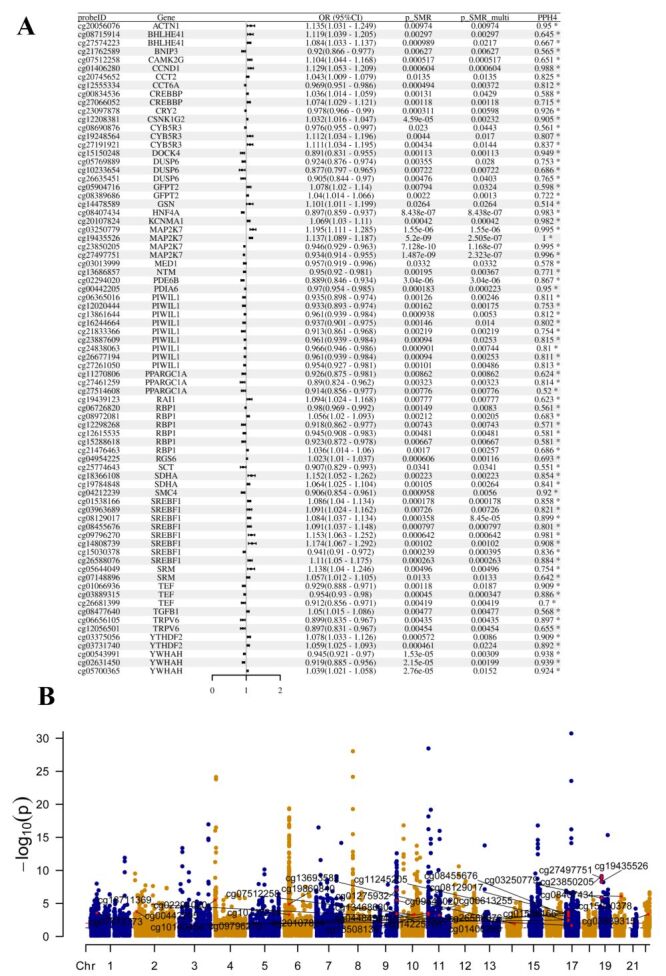
Causal associations between methylation sites of circadian rhythm genes and type 2 diabetes. **Panel A.** Forest plot of SMR analysis results between the GCST90018926 cohort and mQTLs. **Panel B.** Manhattan plot showing the distribution of methylation sites on chromosomes based on SMR analysis results with the GCST90018926 cohort (highlighting key results). mQTL – quantitative trait loci for DNA methylation, SMR – summary-databased Mendelian randomization.

Methylation quantitative trait loci (mQTLs) can help identify genetic variants that affect gene expression or function by altering DNA methylation patterns. These variants may indirectly affect gene expression by modulating the activity of promoters or other regulatory elements without directly altering the coding region. Based on the criteria of *P*-SMR-multi <0.05, *P*-SMR<0.05, and *P*-HEIDI>0.01, we identified a total of 276 methylation sites (corresponding to 125 genes) (Table S2 in the **Online Supplementary Document**). Among these identified features, 77 sites (corresponding to 37 genes) had strong evidence supporting colocalisation (PPH4 > 0.5) (**Figure 2**, Panel A). For example, high methylation levels at the Cytosine-Phosphate-Guanine (CpG) site cg07512258 in the (Calcium/Calmodulin Dependent Protein Kinase II Gamma) CAMK2G gene were positively associated with the risk of T2DM, with an odds ratio (OR) = 1.104 (95% confidence interval (CI) = 1.044–1.168; PPH4 = 0.506). When PPH4 > 0.8 is used as a criterion, there are 44 loci (corresponding to 21 genes) with strong evidence in favour of colocalisation. For different methylation sites within the same gene, the direction of effect might not be consistent. For instance, out of eight methylation sites in the Actinin Alpha 1 (ACTN1)gene, higher methylation levels at cg01847441 and cg11724590 were negatively associated with the risk of T2DM, while higher methylation levels at cg00841968, cg09421468, cg13577778, cg13693582, cg20056076, and cg23052861 were positively associated with the risk of T2DM (Table S2 in the **Online Supplementary Document**). **Figure 2**, Panel B presents the distribution of methylation sites across chromosomes using a Manhattan plot. However, a total of 7814 SNPs were not included in the analysis of mQTLs with the GCST90018926 cohort (Table S3 in the **Online Supplementary Document**).

Additionally, methylation sites corresponding to 44 genes were validated in the FinnGen_R10_T2D cohort (**Table 1**), including Actinin Alpha 1 (ACTN1), Aldolase, Fructose-Bisphosphate A (ALDOA), Calcium/Calmodulin Dependent Protein Kinase II Gamma (CAMK2G), Cyclin D1(CCND1), Chaperonin Containing TCP1 Subunit 2 (CCT2), etc. Methylation sites corresponding to seven genes were validated in the FinnGen_R10_CHARCOT cohort (**Table 1**), including CREB Binding Protein (CREBBP), GNAS Complex Locus (GNAS), Protein Disulfide Isomerase Family A Member 6 (PDIA6), Tumor Necrosis Factor (TNF), Tubulin Beta 1 Class VI (TUBB), Ubiquitin Specific Peptidase 46 (USP46), and Zinc Finger Protein 44 (ZNF44). The complete analysis content from the SMR analysis results for the FinnGen_R10_T2D and FinnGen_R10_CHARCOT cohorts can be found in Table S4 in the **Online Supplementary Document**. And a total of 5512 SNPs were not included in the analysis of mQTLs with the FinnGen cohort (Table S5 in the **Online Supplementary Document**).

**Table 1:** Replication SMR analysis results of mQTLs with the FinnGen data set

GWAS ID	probeID	SYMBOL	*P*_SMR	*P* _SMR_multi	FDR_SMR	OR_SMR	95% CI_SMR
finngen_R10_T2D	cg00841968	ACTN1	0.02071294	0.02071294	0.051502445	1.084	1.012–1.16
finngen_R10_T2D	cg01847441	ACTN1	0.005853644	0.005853644	0.024113519	0.949	0.914–0.985
finngen_R10_T2D	cg09421468	ACTN1	0.02466043	0.02466043	0.057968303	1.104	1.013–1.203
finngen_R10_T2D	cg11724590	ACTN1	0.0147271	0.0147271	0.039083458	0.92	0.86–0.984
finngen_R10_T2D	cg13577778	ACTN1	0.002643993	0.002643993	0.01236851	1.125	1.042–1.214
finngen_R10_T2D	cg20056076	ACTN1	0.000637067	0.000637067	0.0053282	1.152	1.062–1.25
finngen_R10_T2D	cg23052861	ACTN1	1.34E-06	1.34E-06	3.41E-05	1.153	1.088–1.221
finngen_R10_T2D	cg07742718	ALDOA	0.006089353	0.01443082	0.024715609	1.11	1.03–1.196
finngen_R10_T2D	cg07512258	CAMK2G	9.30E-05	9.30E-05	0.001116502	1.109	1.053–1.168
finngen_R10_T2D	cg15192041	CCND1	0.03801291	0.03801291	0.080704332	0.967	0.936–0.998
finngen_R10_T2D	cg20745652	CCT2	0.00342124	0.00342124	0.015479709	1.053	1.017–1.09
finngen_R10_T2D	cg13753209	CLTC	0.04937778	0.04937778	0.100950128	1.057	1–1.116
finngen_R10_T2D	cg07511668	CPT1A	5.31E-07	2.25E-06	2.59E-05	0.957	0.941–0.974
finngen_R10_T2D	cg10879867	CPT1A	2.29E-06	2.29E-06	4.51E-05	0.921	0.89–0.953
finngen_R10_T2D	cg18350739	CPT1A	7.66E-05	7.66E-05	0.000961448	1.11	1.054–1.169
finngen_R10_T2D	cg24488311	CPT1A	9.78E-06	9.78E-06	0.00016867	0.898	0.856–0.942
finngen_R10_T2D	cg11323439	CREB3L1	0.001245884	0.001245884	0.007163833	1.155	1.058–1.261
finngen_R10_T2D	cg00834536	CREBBP	0.01430977	0.011387	0.038553288	1.028	1.006–1.051
finngen_R10_T2D	cg04993286	CREBBP	0.004077822	0.004077822	0.018152885	0.893	0.826–0.965
finngen_R10_T2D	cg27066052	CREBBP	0.01052613	0.01052613	0.032280807	1.058	1.013–1.104
finngen_R10_T2D	cg21473814	CRTC1	0.000949632	0.005327565	0.006535289	1.103	1.041–1.168
finngen_R10_T2D	cg15812322	CRY2	0.000970822	0.000970822	0.006535289	0.94	0.906–0.975
finngen_R10_T2D	cg22234194	CRY2	0.001493876	0.001493876	0.00777943	1.089	1.033–1.149
finngen_R10_T2D	cg23097878	CRY2	5.64E-07	1.11E-07	2.59E-05	0.971	0.96–0.982
finngen_R10_T2D	cg24354272	CRY2	0.001169254	0.001169254	0.007163833	1.073	1.028–1.12
finngen_R10_T2D	cg08690876	CYB5R3	3.93E-06	0.000108385	7.23E-05	0.951	0.931–0.971
finngen_R10_T2D	cg01719718	DCLK1	0.01438764	0.01438764	0.038553288	1.086	1.016–1.159
finngen_R10_T2D	cg04156940	EGFR	0.02433483	0.02433483	0.057900113	0.938	0.887–0.992
finngen_R10_T2D	cg26503877	ETS1	0.001399263	0.006202205	0.007426857	1.061	1.023–1.101
finngen_R10_T2D	cg26559804	ETS1	0.001235363	0.01051693	0.007163833	1.049	1.019–1.08
finngen_R10_T2D	cg26378201	GH1	0.02365584	0.02365584	0.057272034	1.06	1.008–1.115
finngen_R10_T2D	cg06065549	GNAS	0.005692586	0.005692586	0.024113519	1.133	1.037–1.238
finngen_R10_T2D	cg10797197	GNAS	4.31E-05	0.001178714	0.000660741	0.934	0.904–0.965
finngen_R10_T2D	cg14962007	HABP2	0.04552156	0.04552156	0.094465794	1.045	1.001–1.091
finngen_R10_T2D	cg27596495	HCRTR2	0.01639362	0.01639362	0.042286347	0.928	0.873–0.986
finngen_R10_T2D	cg18814314	HSF1	1.96E-06	8.01E-05	4.22E-05	0.966	0.953–0.98
finngen_R10_T2D	cg06331862	IGF1R	0.03753384	0.03753384	0.080704332	1.064	1.004–1.127
finngen_R10_T2D	cg15903819	IGF1R	0.03022983	0.03022983	0.067832789	0.948	0.903–0.995
finngen_R10_T2D	cg00613255	INS	5.52E-11	3.56E-12	1.52E-08	0.855	0.816–0.896
finngen_R10_T2D	cg02613624	INS-IGF2	0.001196522	0.001196522	0.007163833	1.08	1.031–1.132
finngen_R10_T2D	cg04993286	CREBBP	0.004077822	0.004077822	0.018152885	0.893	0.826–0.965
finngen_R10_T2D	cg27066052	CREBBP	0.01052613	0.01052613	0.032280807	1.058	1.013–1.104
finngen_R10_T2D	cg21473814	CRTC1	0.000949632	0.005327565	0.006535289	1.103	1.041–1.168
finngen_R10_T2D	cg15812322	CRY2	0.000970822	0.000970822	0.006535289	0.94	0.906–0.975
finngen_R10_T2D	cg22234194	CRY2	0.001493876	0.001493876	0.00777943	1.089	1.033–1.149
finngen_R10_T2D	cg23097878	CRY2	5.64E-07	1.11E-07	2.59E-05	0.971	0.96–0.982
finngen_R10_T2D	cg24354272	CRY2	0.001169254	0.001169254	0.007163833	1.073	1.028–1.12
finngen_R10_T2D	cg08690876	CYB5R3	3.93E-06	0.000108385	7.23E-05	0.951	0.931–0.971
finngen_R10_T2D	cg01719718	DCLK1	0.01438764	0.01438764	0.038553288	1.086	1.016–1.159
finngen_R10_T2D	cg04156940	EGFR	0.02433483	0.02433483	0.057900113	0.938	0.887–0.992
finngen_R10_T2D	cg26503877	ETS1	0.001399263	0.006202205	0.007426857	1.061	1.023–1.101
finngen_R10_T2D	cg26559804	ETS1	0.001235363	0.01051693	0.007163833	1.049	1.019–1.08
finngen_R10_T2D	cg26378201	GH1	0.02365584	0.02365584	0.057272034	1.06	1.008–1.115
finngen_R10_T2D	cg06065549	GNAS	0.005692586	0.005692586	0.024113519	1.133	1.037–1.238
finngen_R10_T2D	cg10797197	GNAS	4.31E-05	0.001178714	0.000660741	0.934	0.904–0.965
finngen_R10_T2D	cg14962007	HABP2	0.04552156	0.04552156	0.094465794	1.045	1.001–1.091
finngen_R10_T2D	cg27596495	HCRTR2	0.01639362	0.01639362	0.042286347	0.928	0.873–0.986
finngen_R10_T2D	cg18814314	HSF1	1.96E-06	8.01E-05	4.22E-05	0.966	0.953–0.98
finngen_R10_T2D	cg06331862	IGF1R	0.03753384	0.03753384	0.080704332	1.064	1.004–1.127
finngen_R10_T2D	cg15903819	IGF1R	0.03022983	0.03022983	0.067832789	0.948	0.903–0.995
finngen_R10_T2D	cg00613255	INS	5.52E-11	3.56E-12	1.52E-08	0.855	0.816–0.896
finngen_R10_T2D	cg02613624	INS-IGF2	0.001196522	0.001196522	0.007163833	1.08	1.031–1.132
finngen_R10_T2D	cg07096953	INS-IGF2	0.001221641	0.001221641	0.007163833	1.071	1.027–1.116
finngen_R10_T2D	cg12528452	INS-IGF2	0.001216287	0.001216287	0.007163833	1.111	1.042–1.184
finngen_R10_T2D	cg03250779	MAP2K7	9.27E-07	9.27E-07	2.84E-05	1.216	1.124–1.314
finngen_R10_T2D	cg03013999	MED1	0.008544593	0.008544593	0.029452137	0.955	0.923–0.988
finngen_R10_T2D	cg05340686	NFE2L1	0.000723838	0.001102202	0.005549423	0.941	0.909–0.975
finngen_R10_T2D	cg14225168	NOTCH1	0.006864438	0.006864438	0.026684294	1.047	1.013–1.083
finngen_R10_T2D	cg15304464	OPCML	0.03196107	0.003932032	0.071139156	1.047	1.004–1.091
finngen_R10_T2D	cg12926341	PCBP1	0.000706569	0.000706569	0.005549423	0.881	0.818–0.948
finngen_R10_T2D	cg03323696	PDE4D	0.009481468	0.01495394	0.031913234	1.042	1.01–1.075
finngen_R10_T2D	cg06469229	PDE4D	0.007277941	0.001582726	0.02728712	1.022	1.006–1.039
finngen_R10_T2D	cg07330169	PDE4D	0.01124655	0.01124655	0.03373965	1.081	1.018–1.147
finngen_R10_T2D	cg08554295	PDE4D	0.009911665	0.009911665	0.032268893	1.062	1.015–1.113
finngen_R10_T2D	cg11258089	PDE4D	0.01430285	0.01430285	0.038553288	1.089	1.017–1.165
finngen_R10_T2D	cg12400680	PDE4D	0.008024345	0.008024345	0.028393836	1.048	1.012–1.085
finngen_R10_T2D	cg17010100	PDE4D	0.007255279	0.003482024	0.02728712	1.03	1.008–1.052
finngen_R10_T2D	cg22706610	PDE4D	0.01052635	0.01052635	0.032280807	1.055	1.013–1.099
finngen_R10_T2D	cg23094665	PDE4D	0.01357445	0.01357445	0.038553288	1.091	1.018–1.17
finngen_R10_T2D	cg26870744	PDE4D	0.0100548	0.0100548	0.032268893	1.064	1.015–1.115
finngen_R10_T2D	cg02294020	PDE6B	0.01594466	0.01594466	0.041516285	0.938	0.89–0.988
finngen_R10_T2D	cg00442205	PDIA6	0.00089074	0.00043451	0.006303701	0.975	0.96–0.99
finngen_R10_T2D	cg01930746	PDIA6	0.00057639	0.00057639	0.005131731	1.112	1.047–1.18
finngen_R10_T2D	cg04351776	PDIA6	0.000164933	4.33E-05	0.001750828	1.028	1.014–1.044
finngen_R10_T2D	cg08803791	PDIA6	0.00014904	5.58E-05	0.001645405	1.09	1.043–1.14
finngen_R10_T2D	cg15705551	PDIA6	0.002159981	0.002159981	0.010458855	0.882	0.814–0.956
finngen_R10_T2D	cg18436444	PDIA6	0.001333638	0.001333638	0.007352759	1.142	1.053–1.238
finngen_R10_T2D	cg06365016	PIWIL1	0.006795773	0.02530969	0.026684294	0.951	0.917–0.986
finngen_R10_T2D	cg12020444	PIWIL1	0.01391169	0.02656079	0.038553288	0.95	0.913–0.99
finngen_R10_T2D	cg13861644	PIWIL1	0.007804115	0.01268066	0.02834126	0.973	0.954–0.993
finngen_R10_T2D	cg21833366	PIWIL1	0.02851412	0.02851412	0.064507353	0.943	0.894–0.994
finngen_R10_T2D	cg23887609	PIWIL1	0.007782201	0.01398931	0.02834126	0.974	0.954–0.993
finngen_R10_T2D	cg24838063	PIWIL1	0.005660722	0.01536733	0.024113519	0.975	0.957–0.993
finngen_R10_T2D	cg26677194	PIWIL1	0.005790977	0.006039105	0.024113519	0.971	0.951–0.992
finngen_R10_T2D	cg11665562	PSMC1	0.002139315	0.002139315	0.010458855	0.91	0.857–0.966
finngen_R10_T2D	cg14519717	PSMC1	0.02199734	0.02199734	0.053728016	1.092	1.013–1.177
finngen_R10_T2D	cg20036770	PSMC1	0.001030915	0.001030915	0.006774584	0.911	0.862–0.963
finngen_R10_T2D	cg20072951	PSMC1	0.001893459	0.001893459	0.009501722	1.098	1.035–1.165
finngen_R10_T2D	cg20276874	PSMC1	0.001696791	0.001696791	0.008672487	1.09	1.033–1.15
finngen_R10_T2D	cg07469792	RASSF8	0.001293106	0.001293106	0.007283617	0.921	0.876–0.968
finngen_R10_T2D	cg01275932	SEC16A	5.67E-05	5.67E-05	0.000745234	0.88	0.827–0.936
finngen_R10_T2D	cg26123763	SIAH2	0.001358662	7.65E-05	0.007352759	0.944	0.912–0.978
finngen_R10_T2D	cg04212239	SMC4	0.000514578	0.001547285	0.004734113	0.9	0.848–0.955
finngen_R10_T2D	cg01538166	SREBF1	0.01687897	0.01687897	0.043135146	1.048	1.008–1.089
finngen_R10_T2D	cg08129017	SREBF1	0.01031388	0.0136284	0.032280807	1.05	1.012–1.09
finngen_R10_T2D	cg08455676	SREBF1	0.01045817	0.01045817	0.032280807	1.056	1.013–1.101
finngen_R10_T2D	cg09796270	SREBF1	0.0209542	0.0209542	0.051637136	1.079	1.012–1.152
finngen_R10_T2D	cg14808739	SREBF1	0.01833465	0.01833465	0.046003304	1.092	1.015–1.174
finngen_R10_T2D	cg15030378	SREBF1	0.008643562	0.01877322	0.029452137	0.963	0.937 - 0.991
finngen_R10_T2D	cg26588076	SREBF1	0.01301742	0.01301742	0.037425083	1.059	1.012–1.108
finngen_R10_T2D	cg05644049	SRM	0.03611602	0.03611602	0.078488358	1.088	1.005–1.178
finngen_R10_T2D	cg07148896	SRM	0.01000846	0.01000846	0.032268893	1.059	1.014–1.106
finngen_R10_T2D	cg02156899	TBL3	0.01121475	0.02165176	0.03373965	0.974	0.955–0.994
finngen_R10_T2D	cg06799756	TBL3	0.0140036	0.0140036	0.038553288	0.95	0.911–0.99
finngen_R10_T2D	cg01066936	TEF	0.002471083	0.01189587	0.011758947	0.931	0.888–0.975
finngen_R10_T2D	cg03889315	TEF	0.007949084	0.03514904	0.028393836	0.964	0.939–0.991
finngen_R10_T2D	cg26681399	TEF	0.03497114	0.03497114	0.07660345	0.933	0.875–0.995
finngen_R10_T2D	cg03375056	YTHDF2	0.000611152	0.006008894	0.005271185	1.075	1.031–1.12
finngen_R10_T2D	cg03731740	YTHDF2	0.000459864	0.008983931	0.004663273	1.056	1.024–1.089
finngen_R10_T2D	cg07540652	ZNF704	0.02478355	0.02478355	0.057968303	1.074	1.009–1.143
finngen_R10_T2D	cg22454686	ZNF704	0.02743241	0.02743241	0.063094543	1.087	1.009–1.171
FinnGen_R10_CHARCOT	cg04993286	CREBBP	0.03335038	0.03335038	0.715888588	0.466	0.23–0.941
FinnGen_R10_CHARCOT	cg00732970	GNAS	0.03371939	0.03371939	0.715888588	0.477	0.241–0.945
FinnGen_R10_CHARCOT	cg06065549	GNAS	0.04714929	0.04714929	0.733288641	0.447	0.202–0.99
FinnGen_R10_CHARCOT	cg13809528	PDIA6	0.04425996	0.04425996	0.733288641	0.638	0.412–0.989
FinnGen_R10_CHARCOT	cg10717214	TNF	0.001004226	0.001004226	0.138583188	3.821	1.719–8.492
FinnGen_R10_CHARCOT	cg19110902	TUBB	0.001956304	0.001956304	0.179979968	2.785	1.456–5.327
FinnGen_R10_CHARCOT	cg02589501	USP46	0.03992163	0.03992163	0.733288641	1.343	1.014–1.78
FinnGen_R10_CHARCOT	cg01418618	ZNF44	0.01204267	0.02546859	0.62304562	1.413	1.079–1.851
FinnGen_R10_CHARCOT	cg06860823	ZNF44	0.01690433	0.01690433	0.666513583	1.879	1.12–3.152
FinnGen_R10_CHARCOT	cg27506810	ZNF44	0.01354447	0.04231748	0.62304562	0.651	0.463–0.915

CI – confidence interval, FDR – false discovery rate, GWAS – genome-wide association study, mQTL – quantitative trait loci for DNA methylation, OR – odds ratio, SMR – summary-databased Mendelian randomization

### Circadian rhythm-related gene expression and T2DM

**Figure 3:**
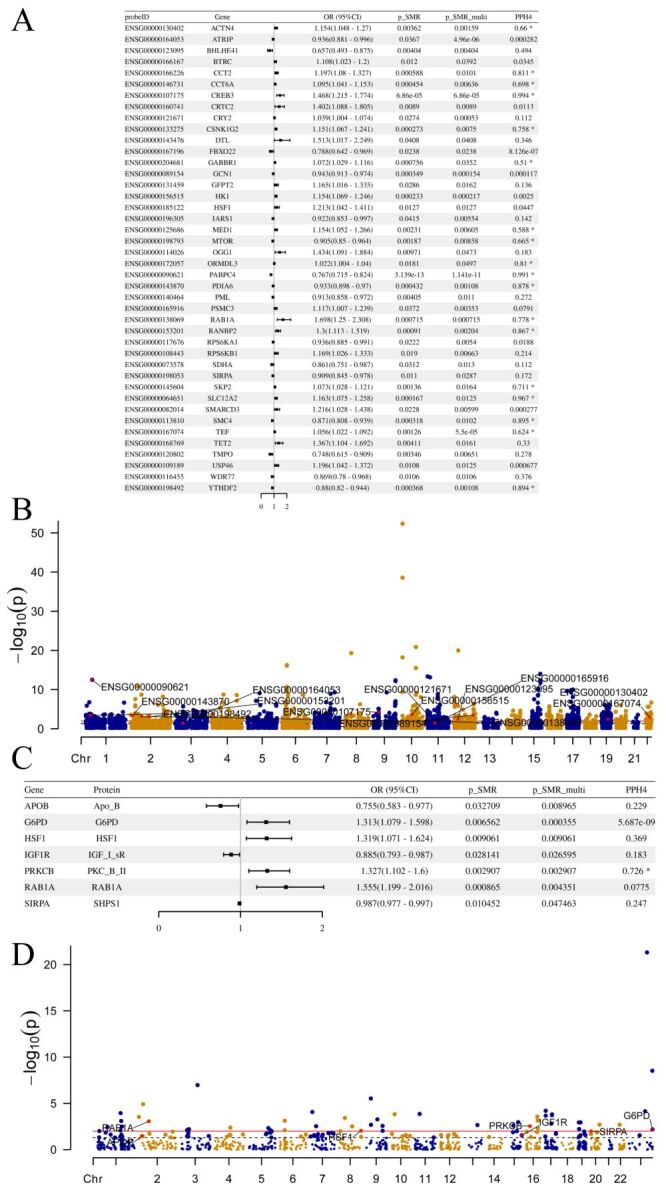
Causal associations between circadian rhythm gene and protein expression and type 2 diabetes. **Panel A.** Forest plot of SMR analysis results between the GCST90018926 cohort and eQTLs. **Panel B.** Manhattan plot of SMR analysis results between the GCST90018926 cohort and eQTLs. **Panel C.** Forest plot of SMR analysis results between the GCST90018926 cohort and eQTLs. **Panel D.** Manhattan plot of SMR analysis results between the GCST90018926 cohort and eQTLs. eQTL – quantitative trait loci for gene expression, SMR – summary-databased Mendelian randomization.

Expression quantitative trait loci (eQTL) can directly demonstrate which genetic variants affect the expression levels of specific genes, thus helping to understand the potential role of these genes in disease development. The causal relationship between circadian rhythm gene expression and T2DM is presented in **Figure 3** and Table S6 in the **Online Supplementary Document**. The complete results of the SMR analysis can be found in Table S7 in the **Online Supplementary Document****.** We identified a total of 42 genes associated with T2DM. Colocalisation analysis revealed that 18 genes had loci associated with T2DM within the colocalisation region of the corresponding SNPs (PPH4 > 0.5) (**Figure 3**, Panel A). And if PPH4 > 0.8 is used as a criterion, there are nine genes with T2DM-related loci located within the colocalised regions of the corresponding SNPs. In addition, there were 17 091 SNPs that were not included in the analysis of eQTLs with the GCST90018926 cohort (Table S8 in the **Online Supplementary Document**).

Furthermore, 17 genes associated with T2DM risk were validated in the FinnGen_R10_T2D cohort (**Table 2**). One gene (Gamma-Aminobutyric Acid Type B Receptor Subunit 1, GABBR1) associated with T2DM risk was validated in the FinnGen_R10_CHARCOT cohort (**Table 2**). The complete results of the SMR analysis for the FinnGen_R10_T2D and FinnGen_R10_CHARCOT cohorts can be found in Table S9 in the **Online Supplementary Document**. Also, 11 258 SNPs were not included in the analysis of eQTLs with the FinGen cohort (Table S10 in the **Online Supplementary Document**).

**Table 2:** Replication SMR analysis results of eQTLs with the FinnGen data set

GWAS ID	probeID	SYMBOL	*P*_SMR	*P* _SMR_multi	FDR_SMR	OR_SMR	95% CI_SMR
Finngen_R10_T2D	ENSG00000123095	BHLHE41	0.006061085	0.006061085	0.015813301	0.71	0.556–0.907
Finngen_R10_T2D	ENSG00000166167	BTRC	0.006200044	0.03303018	0.015813301	1.166	1.045–1.302
Finngen_R10_T2D	ENSG00000107175	CREB3	0.001230061	0.001230061	0.005166256	1.368	1.131–1.655
Finngen_R10_T2D	ENSG00000167196	FBXO22	0.03484675	0.03484675	0.066525614	0.8	0.65–0.984
Finngen_R10_T2D	ENSG00000089154	GCN1	0.004412802	3.51E-07	0.013238406	0.951	0.919–0.985
Finngen_R10_T2D	ENSG00000185122	HSF1	3.18E-05	3.18E-05	0.000334166	1.453	1.218–1.732
Finngen_R10_T2D	ENSG00000125686	MED1	0.01219264	0.01986624	0.026952152	1.14	1.029–1.262
Finngen_R10_T2D	ENSG00000090621	PABPC4	2.80E-11	3.06E-09	1.17E-09	0.775	0.72–0.836
Finngen_R10_T2D	ENSG00000143870	PDIA6	3.36E-06	0.000288976	4.70E-05	0.909	0.873–0.946
Finngen_R10_T2D	ENSG00000138069	RAB1A	0.04779491	0.04779491	0.084696378	1.353	1.003–1.826
Finngen_R10_T2D	ENSG00000153201	RANBP2	0.001175199	0.02199958	0.005166256	1.229	1.085–1.391
Finngen_R10_T2D	ENSG00000117676	RPS6KA1	0.002947808	0.000154541	0.009523687	0.911	0.856–0.969
Finngen_R10_T2D	ENSG00000082014	SMARCD3	0.007960595	0.001065981	0.018574722	1.162	1.04–1.298
Finngen_R10_T2D	ENSG00000113810	SMC4	0.000140907	0.004154551	0.001183619	0.864	0.802–0.932
Finngen_R10_T2D	ENSG00000168769	TET2	0.001401742	0.006077988	0.005352106	1.42	1.145–1.76
Finngen_R10_T2D	ENSG00000116455	WDR77	0.04839793	0.04839793	0.084696378	0.894	0.799–0.999
Finngen_R10_T2D	ENSG00000198492	YTHDF2	0.00042873	0.002333117	0.002944721	0.886	0.828–0.948
finngen_R10_CHARCOT	ENSG00000204681	GABBR1	0.004029425	0.0374479	0.16923585	3.746	1.523–9.216

CI – confidence interval, eQTL – quantitative trait loci for gene expression, FDR – false discovery rate, GWAS – genome-wide association study, OR – odds ratio, SMR – summary-databased Mendelian randomization

### Circadian rhythm-related protein abundance and T2DM

The causal relationship between circadian rhythm-related protein abundance and T2DM is presented in **Figure 3**, Panels C–D and Table S11 in the **Online Supplementary Document**. The complete results of the SMR analysis can be found in Table S12 in the **Online Supplementary Document**. Using the criteria *P*-SMR-multi <0.05, *P*-SMR<0.05 and *P*-HEIDI>0.01, we identified seven proteins (Apolipoprotein B (APOB), Glucose-6-Phosphate Dehydrogenase (G6PD), Heat Shock Transcription Factor 1(HSF1), Insulin Like Growth Factor 1 Receptor (IGF1R), Protein Kinase C Beta (PRKCB), Ras-related protein Rab-1A (RAB1A), Signal Regulatory Protein alpha (SIRPA)) corresponding to genes associated with T2DM. Among these, PRKCB had strong colocalisation evidence (PPH4 > 0.5) (**Figure 3**, Panel C). There was no evidence of colocalisation of protein levels if PPH4 > 0.8 was used as a criterion. However, in the analysis of pQTLs with the GCST90018926 cohort, a total of 23 074 SNPs were not included (Table S13 in the **Online Supplementary Document**).

Among these, HSF1 and RAB1A were validated in the FinnGen_R10_T2D cohort (**Table 3**). However, the results from the discovery cohort (GCST90018926) were not validated in the FinnGen_R10_CHARCOT cohort. The complete results of the SMR analysis for the FinnGen_R10_T2D and FinnGen_R10_CHARCOT cohorts can be found in Table S14 in the **Online Supplementary Document**. And, there were a total of 11 117 SNPs that were not included in the analysis of pQTLs with the FinGen cohort (Table S15 in the **Online Supplementary Document**).

**Table 3:** Replication SMR analysis results of pQTLs with the FinnGen data set

GWAS ID	probeID	*P* _SMR	*P* _SMR_multi	FDR_SMR	OR_SMR	95% CI_SMR
Finngen_R10_T2D	HSF1	3.34E-05	3.34E-05	0.000200443	1.656	1.305–2.101
Finngen_R10_T2D	RAB1A	0.000688002	0.003106622	0.002064007	1.611	1.223–2.122

CI – confidence interval, FDR – false discovery rate, GWAS – genome-wide association study, OR–odds ratio, pQTL – quantitative trait loci for protein expression, SMR – summary-databased Mendelian randomization

### Integrating evidence from multi-omics levels

Based on key results from T2DM and circadian rhythm-related mQTLs and eQTLs, we further explored the regulation of blood methylation on the expression of key circadian rhythm genes in T2DM through SMR analysis integrating mQTL and eQTLs data. Using the criteria *P*-SMR-multi <0.05, *P*-SMR<0.05 and *P*-HEIDI>0.01, we identified 20 methylation sites for 13 genes (Basic Helix-Loop-Helix Family Member E41(BHLHE41), CREB Regulated Transcription Coactivator 2(CRTC2), Cryptochrome Circadian Regulator 2 (CRY2), Gamma-Aminobutyric Acid Type B Receptor Subunit 1 (GABBR1), Mediator Complex Subunit 1(MED1), Mechanistic Target Of Rapamycin Kinase (MTOR), ORMDL Sphingolipid Biosynthesis Regulator 3 (ORMDL3), Protein Disulfide Isomerase Family A Member 6 (PDIA6), Proteasome 26S Subunit, ATPase 3 (PSMC3), Structural Maintenance Of Chromosomes 4 (SMC4), Transcription Elongation Factor (TEF), Ubiquitin Specific Peptidase 46 (USP46), YTH N6-methyladenosine RNA binding protein 2 (YTHDF2)) that may play crucial roles in T2DM (Table S16 in the **Online Supplementary Document**). The complete results can be found in Table S17 in the **Online Supplementary Document**. However, no associations were found when integrating eQTLs and pQTLs data.

By integrating the SMR analysis results from blood mQTLs, eQTLs, and pQTLs, we identified one intersecting gene (HSF1) that may have a causal association with T2DM (Table S18 in the **Online Supplementary Document**). In the mQTLs data, the methylation site corresponding to HSF1 is cg18814314, with an OR = 0.981 (95% CI = 0.968–0.994; FDR-adjusted *P* = 0.1180), which is negatively associated with the risk of T2DM. In the eQTLs data, HSF1 is positively associated with the risk of T2DM, with an OR = 1.213 (95% CI = 1.042–1.411; FDR-adjusted *P* = 0.1656). In the pQTLs data, HSF1 is also positively associated with the risk of T2DM, with an OR = 1.319 (95% CI = 1.071–1.624; FDR-adjusted *P* = 0.1858). Thus, a hypothetical mechanism may exist where lower methylation levels of cg18814314 up-regulate HSF1 gene expression, leading to higher HSF1 protein expression and thereby increasing the risk of T2DM. The ProbeID corresponding to HSF1 is ENSG00000185122. Then, we used locus zoom plots to show the distribution of HSF1 (eQTL and mQTL) and used SMREffectPlot to display their effects on T2DM risk (Figure S1 in the **Online Supplementary Document**).

## DISCUSSION

### Key findings and novel insights

Using an integrated Multi-omics Mendelian Randomization (MMR) approach, our study systematically investigates the potential pathogenic gene associations between circadian rhythm regulation and T2DM. Through SMR and colocalisation screening (*P*-SMR-multi <0.05, *P*-SMR<0.05, and *P*-HEIDI>0.01), we identified 47 mQTLs, five eQTLs, and three pQTLs related to circadian rhythms that showed causal relationships with disease outcomes in the discovery stage. By integrating multi-omics evidence, we were unable to establish a causal association. However, based on the gene names corresponding to each QTL from the SMR analysis results, we identified an intersecting gene, HSF1, which may have a causal relationship with T2DM.

### HSF1 as a key gene regulating the pathogenesis of T2DM through circadian rhythms

At the mQTL level, high methylation of the HSF1-associated site cg18814314 is negatively correlated with T2DM risk. Conversely, in the eQTL and pQTL data, high levels of HSF1 expression are positively correlated with T2DM risk, suggesting that increased HSF1 expression may enhance susceptibility to T2DM. This pattern of expression may reflect the complex role of HSF1 in different layers of circadian regulation and its dual role as a transcription factor in responding to cellular stress, metabolic regulation, and protein homeostasis. DNA methylation predominantly occurs at CpG dinucleotides and is commonly associated with gene silencing [35,36]. High levels of CpG methylation lead to long-term gene silencing by impeding the binding of transcription factors or recruiting chromatin remodelling mediators, among other mechanisms [37,38]. Conversely, low CpG methylation within gene promoter regions establishes a chromatin state permissive to transcription, thereby facilitating gene expression [39]. Therefore, disruption of circadian rhythms may play a significant role in the pathogenesis of T2DM by affecting the normal regulation of HSF1, disturbing the balance of heat shock proteins, promoting insulin resistance, and impairing the function of pancreatic β-cells.

Available studies suggest that HSF1 has multiple roles, both in preventing insulin resistance [40] and in inhibiting glycolipotoxicity-induced β-cell apoptosis [41]. On the one hand, HSF1 enhances cellular adaptation to metabolic stresses, such as oxidative stress and endoplasmic reticulum stress. This helps to improve insulin signalling and reduce the occurrence of insulin resistance. On the other hand, in a glycolipotoxic environment, HSF1 can prevent damage to β-cells by activating Heat Shock Proteins (HSPs) and other protective molecules, thus maintaining their normal function and survival. For T2D, HSF1 may have different effects. For instance, some studies have shown that the expression of HSF1 in the liver and pancreatic tissues of T2D monkeys is completely opposite [42]. Additionally, Li et al. suggest that HSF1 can alleviate the impaired muscle insulin signalling and insulin resistance in high-fat-fed mice [40]. However, another study indicates that the gene polymorphism encoding HSF1 increases the risk of T2D [43]. The SMR analysis strongly indicates that the higher HSF1 expression/protein level predicted by genes is associated with an increased risk of T2DM.

Although direct research evidence is currently insufficient, we hypothesise several possible mechanisms based on related studies to explain the potential causal relationship between HSF1 and T2DM from the perspective of circadian rhythms. First, HSF1, as a key transcription factor, is closely related to circadian rhythm regulation, with its activity and expression levels showing circadian fluctuations, possibly regulated by core circadian rhythm genes such as Basic Helix-Loop-Helix ARNT Like 1(BMAL1) [44,45]. Second, studies have reported that the activation of HSF1 can improve pancreatic islet cell function and metabolic health by increasing the expression of HSPs [46]. Therefore, we hypothesised that circadian rhythm disruption may lead to abnormal activation of HSF1, affecting the expression of HSPs, resulting in insulin resistance and pancreatic islet cell dysfunction. In addition, prolonged metabolic stress may lead to persistent activation of HSF1, which in turn triggers a chronic inflammatory response. Chronic inflammation is an important causative factor in T2DM, which can exacerbate insulin resistance and impair β-cell function. Although HSF1 protects β-cells from damage in the short term, long-term high expression may lead to cellular senescence or dysfunction. Cellular senescence is closely associated with T2DM as it affects insulin secretion and sensitivity. Inflammation plays a key role in the occurrence and development of T2DM. Chronic low-grade inflammation can interfere with insulin signalling, leading to insulin resistance and subsequently triggering T2DM [47]. We believe that in the context of T2DM, the high expression of HSF1 may activate some pro-inflammatory signalling pathways. For instance, HSF1 may promote the expression of inflammatory factors through certain mechanisms [48]. In T2DM patients, the levels of inflammatory factors such as Tumour Necrosis Factor-alpha (TNF-α) and Interleukin-6 (IL-6) are elevated [49]. These inflammatory factors can inhibit the phosphorylation of insulin receptor substrate, disrupt the insulin signalling pathway, and cause insulin resistance [50]. The high expression of HSF1 may exacerbate insulin resistance by enhancing the production of these pro-inflammatory factors, thereby increasing the risk of T2DM. Finally, we believe that the MR findings reflect a gene-level and population-level effect, which may capture the consequences of chronic HSF1 dysregulation rather than an acute protective response.

In this study, we found that HSF1 was consistently associated with T2DM at multiple omics levels, including m/e/pQTL, showing strong biological coherence. To the best of our knowledge, this is the first study to systematically link HSF1 to T2DM risk within the context of circadian rhythm genes using SMR, integrating multi-omics QTL data. As mentioned before, previous studies have mainly highlighted HSF1 as a key regulator of cellular stress response and proteostasis, but its role in metabolic regulation, particularly in glucose homeostasis, has not been widely recognised. Our findings suggest that HSF1 may not only be part of the circadian regulatory network, but also serve as a potential mediator connecting circadian disruption with metabolic dysregulation.

Furthermore, the integrative SMR approach combining mQTL, eQTL, and pQTL data represents a novel analytical strategy in this field. This multi-level causal inference framework enhances the confidence in identifying candidate genes and provides multi-layered evidence for understanding the molecular links between circadian rhythm and T2DM. Such an omics-informed causal inference approach not only improves functional interpretability but also offers prioritised targets for future mechanistic studies and potential therapeutic interventions.

### Multomics integration perspective of other candidate genes

The integration of mQTL and eQTL data in this study reveals 20 circadian rhythm-related methylation sites and 13 genes potentially playing significant roles in T2DM. Interestingly, these genes, such as BHLHE41 [51,52], CRTC2 [53,54], CRY2 [55,56] and TEF [57], are involved in regulating circadian rhythms and various biological processes, including transcription regulation, signal transduction, and metabolic pathways, which are critical in the pathophysiology of T2DM. In contrast, the integration of eQTL and pQTL data did not reveal significant associations. This lack of associations might be due to the more complex genetic regulation of protein expression. Studies have found that protein expression can be influenced by post-transcriptional modifications, protein degradation rates, or other regulatory mechanisms not captured by eQTLs (expression quantitative trait loci) [58,59].

### Methodological considerations and advantages

The mQTL primarily reflects changes in DNA methylation status, a form of epigenetic regulation that typically occurs at cytosine bases on CpG islands [60]. DNA methylation can affect gene accessibility and promoter activity prior to transcription initiation. And mQTL provide information about which genetic variants may affect gene expression or function by altering DNA methylation patterns. These variants can be located in promoter regions, enhancer regions, or other regulatory elements of genes. In contrast, eQTLs directly reflect changes in the level of gene expression [26], which includes multiple steps such as transcription, processing, transport and stability of mRNA. Therefore, eQTL focus more on post-transcriptional regulatory mechanisms. In addition, eQTL provide direct functional evidence of whether and how a specific genetic variant affects the biological function of a target gene. It can reveal splicing variants, the role of non-coding RNAs, protein translation efficiency, and so on. Although DNA methylation is one of the important mechanisms regulating gene expression, it does not always lead directly to expression changes. It is first affected by chromatin status [61]. On the one hand, DNA methylation is usually associated with heterochromatin (tightly packed chromatin), which represses gene expression. However, if the chromatin state of a region is very open (*e.g*. an active promoter), gene expression may not be significantly repressed even in the presence of hypermethylation. On the other hand, the state of chromatin is also affected by histone modifications (*e.g*. acetylation, methylation). Certain histone modifications can counteract the repressive effects of DNA methylation and maintain active gene expression. Second, transcription factors may also affect their expression [62,63]. Transcription factors and other regulatory proteins need to bind to DNA to initiate or enhance transcription. If high methylation of a region does not affect the binding of key transcription factors, then the expression of that gene may not be significantly affected. Alternatively, the synergistic action of multiple transcription factors or cofactors may overcome the effects of a single methylated site. For example, a potent activator may preferentially bind and promote transcription even in the presence of some methylation inhibition.

### Limitations of the study

Our study, through integrated multi-omics analysis, not only encompasses traditional gene expression analysis but also extends to epigenetic and protein expression levels, providing richer information for deciphering the complex pathophysiological mechanisms of T2DM. However, the study has some limitations. First, our analysis is primarily based on summary-level data, which may not capture all complex interactions between genetic and epigenetic factors. This may lead to an incomplete understanding of certain regulatory mechanisms. Second, although our study employs a multi-omics approach, it remains an observational study and cannot fully establish causality. It is therefore not possible to completely exclude the influence of confounding factors or to establish a definitive causal relationship. Third, because these data sets are based on publicly available GWAS pooled results, the possibility of potential sample overlap cannot be completely ruled out, thereby exaggerating effect sizes or reducing statistical validity; future studies could further validate the conclusions by obtaining individual-level data or explicit information on sample sources. Furthermore, as this study is based on GWAS summary data, individual-level environmental or lifestyle data cannot be directly obtained. Gene-environment interactions may have influenced the analysis results and could lead to systematic bias. For instance, circadian rhythm disruption behaviours (such as night shift work) are not only influenced by genetic background but may also, in turn, affect metabolic function. Additionally, there is also the possibility of reverse causality, where T2DM-related metabolic abnormalities may affect the regulatory pathways of the circadian rhythm system. Therefore, the current observed associations may be influenced by complex feedback mechanisms. Future longitudinal cohort time-series studies will further clarify the causal direction and, in combination with environmental exposure data, validate our findings. Although we adopted the Benjamini-Hochberg method for multiple testing correction and selected an appropriate *P*-value threshold, it is still impossible to completely rule out the possibility of false positives. Future studies can further enhance the reliability of the results by increasing the sample size, applying more stringent correction methods, or combining with other experimental verification approaches. Our MR research findings, including the QTLs of HSF1, were replicated in the independent FinnGen data set, which strengthened the statistical evidence for these associations. However, like most MR analyses based on summary data, this study did not provide direct functional validation of the underlying biological mechanisms. Experimental studies (*e.g*. in vitro or in vivo models manipulating HSF1 expression or methylation under circadian rhythm challenges) are needed to confirm the exact functional roles of HSF1 and other identified candidate genes in the pathogenesis of T2DM. And, even though the SMR study showed a potential effect of the circadian gene HSF1 on T2D at the m/e/pQTL level, it cannot be ruled out that HSF1 may also affect T2D through other pathways. Further functional validation studies are needed to support these findings. And the effects of gene-environment interactions can be further explored in future studies with individual-level data. In the future, the understanding of complex interactions can be improved by combining more types of histological data to construct a more complete regulatory network; and designing more rigorous research protocols, including longitudinal cohort studies and bidirectional MR analyses, to enhance the reliability and accuracy of causal inference; steps were also taken to ensure that the GWAS data sets used were from independent population samples, and the results were further validated through sensitivity analyses and cross-validation.

## CONCLUSIONS

In conclusion, this study, through SMR analysis based on aggregated data, identified multiple circadian rhythm-related genes (such as HSF1) that may have a potential causal relationship with T2DM. Future research could combine time-series methylation studies in prediabetic populations or regulate HSF1 expression in cell/animal models to explore its impact on glucose homeostasis under different circadian rhythm conditions, thereby validating its functional mechanism. This study is mainly based on aggregated data from European populations, which has certain population limitations, and the results need to be verified by subsequent functional experiments to clarify the biological pathways.

## Additional material


Online Supplementary Document
