# Supplementary material for: The role of circadian rhythm-related genes in type 2 diabetes from a multi-omics perspective
Source: J Glob Health. 2025 Aug 29;15:04227. doi: 10.7189/jogh.15.04227 (PMC12395209; doi:10.7189/jogh.15.04227)

Supplement to: Xie L, Huang D, Zha X, Wei C, Dong J, Zheng H, Xu Z, Wu J, Lai P. The Role of circadian rhythm-related genes in type 2 diabetes from a multi-omics perspective. J Glob Health. 2025;15:04227.

Supplementary Figure 1. SMR analysis results of HSF1 in eQTL and mQTL. A: SMR Locus Plot of cg18814314 in mQTL.; B: SMR Effect Plot of cg18814314 in mQTL; C: SMR Locus Plot of HSF1 in eQTL; D: SMR Effect Plot of HSF1 in eQTL.

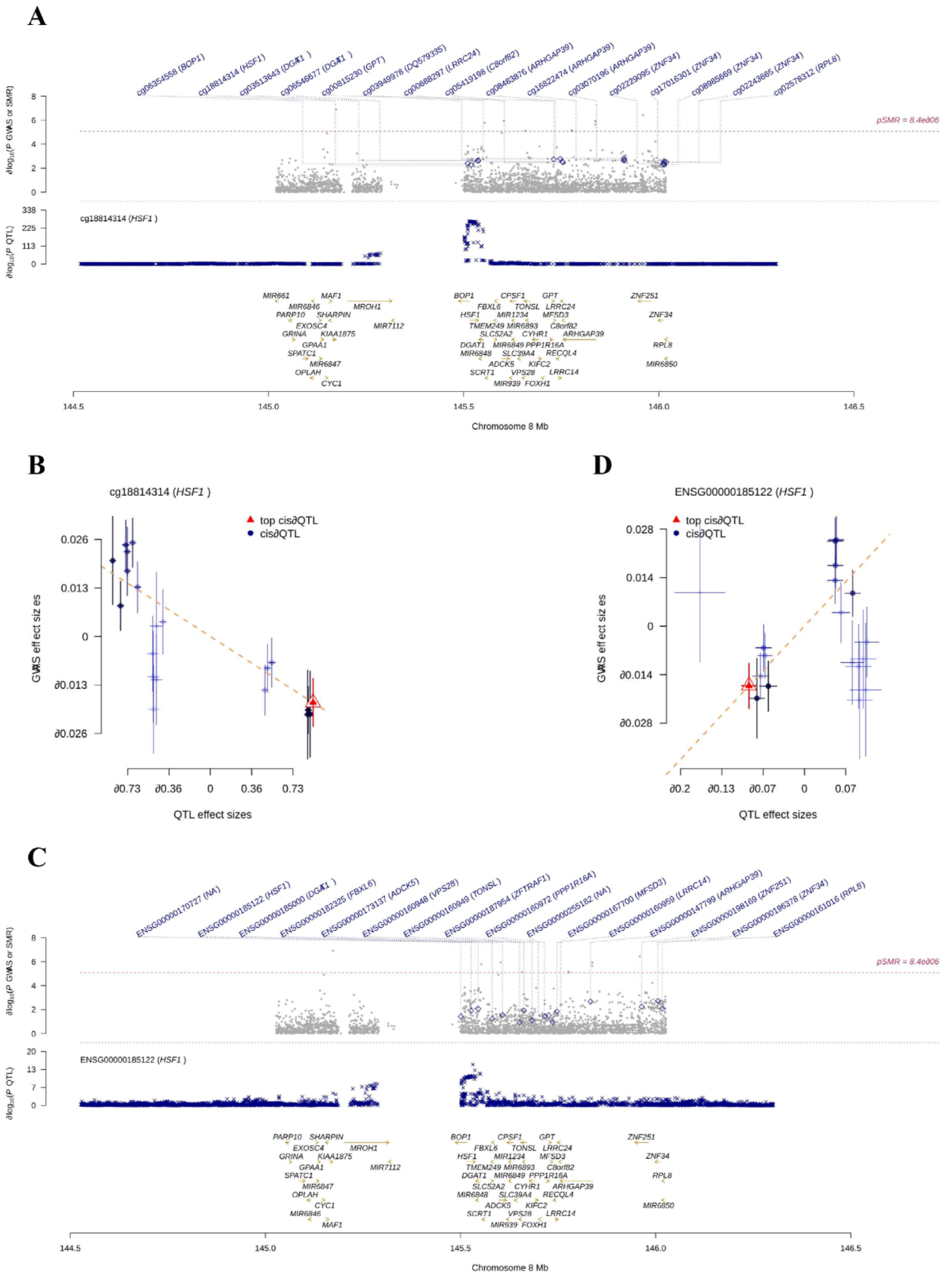

Supplement: Online Supplementary Document [file jogh-15-04227-s001.zip › jogh-15-04227-s001.pdf]
